# Collective resonance: social relationships outperform confidence in explaining exercise-induced happiness in a collectivist society

**DOI:** 10.3389/fpsyg.2026.1887009

**Published:** 2026-08-10

**Authors:** Cheng Fang, Jieyou Zhou

**Affiliations:** 1 Center for Physical Education, Nanfang College Guangzhou, Guangzhou, China; 2 Business School of Sports, Guangzhou Sport University, Guangzhou, China

**Keywords:** collective resonance, confidence, happiness, physical exercise, social relationships

## Abstract

Physical exercise is widely recognized as a key contributor to subjective well-being, yet the pathways underlying exercise-related happiness have largely been framed within individualistic psychological models emphasizing confidence and self-mastery. In collectivist societies, however, physical exercise is frequently embedded in shared social contexts, suggesting that its well-being benefits may rely more heavily on social relational resources. Building on this perspective, this study introduces “collective resonance” as a preliminary interpretative framework to understand the social resources linked to shared physical activity, and examines the respective roles of social relationships and confidence in explaining the association between physical exercise and happiness among Chinese adults. Using data from a nationally representative sample (*N* = 23,059; mean age = 42.50 years, SD = 18.45; 51% male, 49% female), regression and parallel mediation analyses were conducted. The results indicate a significant positive association between regular exercise and happiness (*β* = 0.371, *p* < 0.001). Mediation analyses show that social relationships explain a substantially larger share of this association (25.88%) than individual confidence (11.59%). In addition, the association between exercise and happiness is significantly stronger among rural residents than among urban residents. Overall, the findings underscore the importance of social relational processes in explaining exercise-related happiness in collectivist contexts and suggest that socially facilitated physical activity may be particularly positively associated with well-being.

## 1 Introduction

In the landscape of modern China, happiness is not merely a private psychological state but a deeply embedded social outcome. As the nation undergoes rapid modernization, the traditional social ties that once provided a sense of belonging are increasingly strained by sedentary lifestyles and urban “atomization.” While physical exercise (PE) is a known antidote to psychological stress, current research often focuses on “internal” resources like self-confidence, leaving the “external” social pathways underexplored in non-Western contexts.

Western psychological models, such as Self-Determination Theory (SDT), traditionally posit that exercise fosters happiness by satisfying the need for competence (self-efficacy). However, in a collectivist society where interpersonal harmony is the core of the self-concept, the need for relatedness may take center stage. In China, exercise is rarely a solitary endeavor; it is visible in the rhythmic synchrony of “Square Dancing” (Guangchangwu), the shared focus of morning Tai Chi, and the camaraderie of workplace badminton. These activities transform physical movement into a “social glue.”

This study challenges the “individual-mastery” narrative by asking: Is exercise associated with greater happiness because we feel stronger, or because we feel more connected? By leveraging the 2022 China Family Panel Studies (CFPS), we test a comprehensive model that weighs the internal path (Confidence) against the external path (Social Relationships). We specifically hypothesize that in the Chinese context, social relationships will emerge as a primary mediating pathway. Furthermore, we investigate how this “socially driven happiness” varies across the urban–rural divide, exploring whether exercise acts as a compensatory pathway in rural areas where formal social infrastructure may be lacking. Specifically, this study addresses three research questions: (1) Is physical exercise positively associated with happiness among Chinese adults? (2) Do confidence and social relationships mediate this association? (3) Does the social pathway explain a larger proportion of this association than the individual confidence pathway?

By integrating the Broaden-and-Build Theory with a lifespan and cultural perspective, this research offers “Collective Resonance” as a preliminary interpretative framework to better understand exercise-induced well-being. Unlike general constructs such as a sense of belonging or social support—which describe broad relational states—collective resonance specifically captures the synergistic social energy and shared resources uniquely facilitated by synchronized, group-based physical activity.

## 2 Literature review and research hypotheses

### 2.1 The happiness dividend: exploring the universal link between activity and well-being

A substantial body of research demonstrates that physical exercise (PE) is positively associated with subjective well-being and happiness across different populations and cultural contexts. According to the Broaden-and-Build Theory, positive emotions generated through activities such as exercise broaden individuals’ thought–action repertoires and, over time, help build enduring psychological, social, and physical resources (Fredrickson, 2001). Similarly, Self-Determination Theory (SDT) emphasizes that fulfilling basic psychological needs for competence, autonomy, and relatedness fosters intrinsic motivation and well-being (Ryan and Deci, 2017; Teixeira et al., 2012).

Multiple high-quality meta-analyses provide robust support for this relationship. A meta-analysis of 64 independent samples found that leisure-time physical activity is positively associated with subjective well-being (*r* = 0.12), with the strongest effects on positive affect (Wiese et al., 2018). Another review of 120 experimental and longitudinal studies reported a small-to-moderate beneficial effect of physical activity on subjective well-being (Hedges’ *g* = 0.21), particularly in randomized controlled trials (Buecker et al., 2021). Furthermore, regular physical activity consistently improves quality of life across the adult lifespan (Mahindru et al., 2023).

Evidence in Chinese contexts is equally consistent. A cross-sectional study of 404 Chinese university students using structural equation modeling found that physical activity was positively associated with subjective well-being (Liao et al., 2023). Short-term interventions show that even 4 weeks of regular exercise can significantly improve happiness, life satisfaction, and self-esteem (Iwon et al., 2021). In China, Zhang (2023) found that regular physical activity positively predicts subjective well-being. Based on these findings, we propose:

Hypothesis 1 (*H1*): Physical exercise is positively associated with individuals’ happiness.

### 2.2 The internal engine: confidence as a psychological prerequisite for happiness

Confidence, or individuals’ beliefs in their capacity to succeed (often measured as self-efficacy), is a key psychological resource that influences emotions (Bandura, 1997). Regular physical exercise strengthens confidence through mastery experiences and physiological feedback.

Studies across various cultural contexts demonstrate that self-efficacy, self-esteem, or confidence mediates the exercise–well-being relationship. For instance, recent evidence from semi-collectivist societies, such as Türkiye, highlights the mediating role of self-esteem and self-competence in the correlation between physical activity, parenting styles, and subjective well-being (Demir Kaya and Kaya, 2026; Odacı et al., 2026). Similarly, a 12-month study with American adults showed that improvements in exercise self-efficacy mediated the effect of physical activity on positive affect (White et al., 2018). Among older European adults, self-efficacy has been identified as a mediator between physical activity and emotional well-being (McAuley et al., 2006; Resnick and Inguito, 2011). In Chinese samples, Guo et al. (2024) reported that physical exercise increases self-efficacy and confidence, which in turn enhance subjective well-being among university students. Peng et al. (2023) further linked exercise identity and global self-esteem as parallel mediators. While confidence is an essential internal resource, we hypothesize it acts as a secondary pathway compared to social factors:

Hypothesis 2 (*H2*): Confidence mediates the relationship between physical exercise and happiness.

### 2.3 The cultural heart: why social ties dominate the Chinese happiness equation

High-quality social relationships are one of the strongest and most consistent predictors of happiness across cultures (Diener and Seligman, 2002). In China, exercise frequently occurs in social contexts (e.g., group fitness, square dancing), creating natural opportunities for interaction. A study in the UK demonstrated that well-being benefits are larger when exercise is performed in social settings, with perceived social connection mediating the effect (Haslam et al., 2023). Systematic reviews have also found that social support and belonging explain a significant proportion of the mental health benefits of sport (Eime et al., 2013; Vella et al., 2023).

Evidence from Chinese samples aligns closely. Guo et al. (2024) found that social support mediates the effect of physical activity on well-being among university students. Zhang et al. (2025) observed that social–emotional competencies partially mediate the PE–happiness link. Given the collectivist orientation of Chinese society, we argue that social relationships serve as the primary pathway for the “building” of happiness through exercise:

Hypothesis 3 (*H3*): Social relationships mediate the relationship between physical exercise and happiness and serve as the dominant mediating pathway.

### 2.4 From self-efficacy to social synergy: testing the sequential resource model

Beyond independent mediation, confidence and social relationships may operate sequentially. Higher confidence encourages individuals to seek social interactions, and positive social feedback reinforces confidence and happiness. A systematic review identified a sequence whereby physical activity enhances psychological resources (including self-efficacy), which subsequently promotes social engagement (Vella et al., 2023). A longitudinal study with adolescents similarly showed the path: exercise → self-efficacy → peer support → life satisfaction (Lubans et al., 2018).

In Chinese contexts, Yao et al. (2022) reported a similar pathway in students: physical exercise → perceived social support → exercise self-efficacy → subjective well-being. Guo et al. (2024) also documented a parallel chain (physical activity → social support → self-efficacy → happiness) in university students. However, this chain may be less pronounced in adults, where social roles are more established. We propose:

Hypothesis 4 (*H4*): Confidence and social relationships form a chain-mediating pathway (physical exercise → confidence → social relationships → happiness) between physical exercise and happiness.

**Figure 1:**
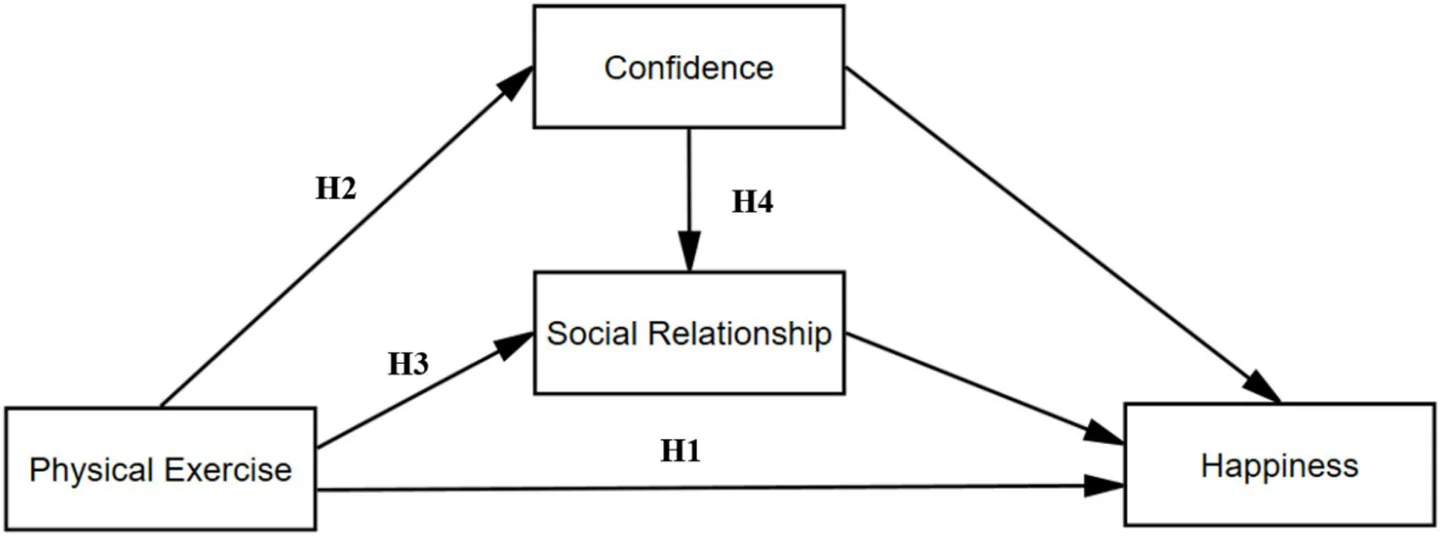
The chain-mediation model of physical exercise on happiness.

The chain-mediation model of physical exercise on happiness is visualized in Figure 1.

### 2.5 Research gap and contribution

While previous literature has established the independent mediating roles of confidence/self-efficacy and social relationships, significant gaps remain in our understanding of how these pathways interact within a collectivist cultural framework.

First, the existing body of evidence is characterized by a “mechanistic silos” approach; few studies have simultaneously tested both mediators in a serial/chain mediation model using large, nationally representative samples—particularly within the Chinese adult population. Most current chain-mediation evidence is limited to adolescent or university student samples. In these younger populations, the sequence from individual confidence to social engagement is often prominent, but it remains unclear if this “confidence-driven” model holds true for adults whose social structures are more institutionalized and stable.

Second, while Western psychological models emphasize the internal resource of self-mastery, there is a lack of empirical research that explicitly weighs these internal psychological paths against external social resources in the Chinese context. By leveraging the 2022 China Family Panel Studies (CFPS) dataset—a nationally representative sample of 23,059 individuals—this study addresses these gaps in the following ways:

#### 2.5.1 Theoretical integration of culture and biology

It integrates major biopsychosocial frameworks—specifically the Broaden-and-Build Theory and Self-Determination Theory—with both global meta-analytic evidence and Chinese-specific cultural findings. This allows for a nuanced exploration of whether the “building” of resources is primarily an individual or a collective process.

#### 2.5.2 A life-span perspective on chain mediation

It empirically tests a comprehensive chain-mediation model (Physical Exercise → Confidence → Social Relationships → Happiness) in a large, adult, nationally representative sample. This provides a vital comparison to adolescent-centric models and clarifies whether adult happiness is explained more by direct social integration than by internal psychological shifts.

#### 2.5.3 Policy-relevant “social baselines”

It provides significant evidence to inform nationwide initiatives. By identifying which pathway—social or psychological—is the dominant correlate of well-being, the study offers empirical support for moving away from individualistic fitness interventions toward socially-embedded, community-based public health strategies aimed at enhancing population happiness.

## 3 Methods

### 3.1 Study population

This study utilized data from the 2022 wave of the China Family Panel Studies (CFPS), a nationally representative, longitudinal survey conducted by the Institute of Social Science Survey (ISSS) at Peking University. All CFPS procedures received ethical approval from the Institutional Review Board of Peking University (Approval/Permit Number: IRB00001052-14010). Written informed consent was obtained from all participants prior to the survey.

The CFPS employs a multi-stage probability sampling method proportional to size, covering 31 provinces, municipalities, and autonomous regions. The survey collects detailed information on demographics, socioeconomic status, health, and well-being, providing a robust dataset for investigating psychological and social outcomes.

The analytical sample was restricted to respondents aged 18 years and older. Cases with missing values on key variables (happiness, physical exercise, confidence, and social relationships) were excluded via listwise deletion. After applying these criteria, the final analytical sample comprised 23,059 individuals. Although listwise deletion may introduce bias if missingness is not completely at random, the proportion of missing data for the core variables was minimal (<5%), reducing the likelihood of substantial bias. This selection ensures that the analysis focuses on adult well-being while maintaining a representative sample.

### 3.2 Variables

#### 3.2.1 Dependent variable

Happiness was measured using a single-item question: “Are you happy?” Responses ranged from 0 (‘very unhappy’) to 10 (‘very happy’), with higher scores indicating greater subjective well-being. Although multi-item scales are generally preferred for latent constructs, single-item measures of global happiness have been validated in large-scale surveys and are considered adequate for capturing general subjective well-being (Diener et al., 2013). This measure provides a concise and interpretable indicator suitable for large population studies, facilitating straightforward comparisons across subgroups.

#### 3.2.2 Core independent variable

Physical exercise (PE) was assessed via the question: “How often do you engage in physical exercise?” Original responses ranged from 1 (‘less than once a month’) to 7 (‘more than twice a day) and 8 (‘never’). Following established practice in major public health cohorts and national surveys that face similarly skewed exercise frequency distributions (e.g., NHANES, UK Biobank, CHARLS; see Bull et al., 2020; Hayes, 2022), we dichotomized the variable into: high-frequency exercisers (≥1 session per week) or low-frequency/non-exercisers (<1 session per week or never).

This cut-off corresponds exactly to the World Health Organization (2020) global recommendation of “at least once per week” as the minimum threshold for deriving mental health benefits and has been adopted in recent high-impact Chinese studies using CFPS data (e.g., Chen et al., 2024). The dichotomization achieves three critical advantages that outweigh the minor loss of granularity in this specific context:

1. It produces clearly balanced groups (38.2% vs. 61.8%), preventing estimation instability common in highly skewed 8-level variables;
2. It directly maps onto actionable public health guidelines, maximizing policy relevance;
3. It avoids violating the parallel-trend/proportionality assumptions required when treating severely skewed 8-level data as continuous or ordinal in linear SEM (Rhemtulla et al., 2012; Flora and Curran, 2004).

#### 3.2.3 Mediating variables

Two mediating variables were included to test the hypothesized pathways linking physical exercise to happiness. Confidence was measured by the item “How confident are you about your future?” with responses ranging from 1 (‘no confidence at all’) to 5 (‘very confident’). Social relationships were measured on an 11-point scale: “How would you rate your relationships with other people?” (0 = lowest, 10 = highest). These variables capture both internal psychological resources and external social resources, aligning with the proposed chain mediation framework.

#### 3.2.4 Control variables

To account for potential confounding factors, the following demographic and socioeconomic variables were included based on prior literature: age (continuous), gender (female = 0, male = 1), marital status (not currently married = 0, married = 1), self-rated health (1 = very healthy to 5 = unhealthy), Hukou (rural = 0, urban = 1), and employment status (not employed = 0, employed = 1). Including these covariates ensures that the estimated associations between physical exercise, mediators, and happiness are not confounded by basic demographic differences.

#### 3.2.5 Reliability, validity, and distribution

Although key constructs were measured with single items, prior research supports their validity in large-scale population surveys (Organization for Economic Co-operation and Development, 2013). The social relationship measure uses an 11-point scale, providing sufficient variance. Skewness and kurtosis values for all key variables fell within acceptable ranges (| < 1|), indicating no severe deviation from normality. To further enhance the robustness of the structural model, the Bollen-Stine bootstrap procedure was applied to adjust fit statistics in the SEM/GSEM analyses, a recommended approach for handling ordinal or non-normal data (Bollen and Stine, 1992).

### 3.3 Data analysis

Analyses were conducted using Stata 16.0. The analytical strategy was designed to systematically evaluate whether the “upward spiral” of happiness is more strongly associated with individual mastery or collective resonance. First, descriptive statistics and Pearson correlations were computed to provide a preliminary overview of the associations between physical exercise (PE), psychological resources, and subjective well-being.

Second, hierarchical multiple linear regression was employed to examine the direct association between PE and happiness. We estimated a series of models to control for demographic and socioeconomic confounding factors, ensuring that the estimated “happiness dividend” of exercise was consistent across different social strata.

Third, we conducted heterogeneity analyses to test the “Social Infrastructure” hypothesis. By stratifying the sample by Hukou status (urban vs. rural), age, and gender, we explored whether exercise functions as a compensatory pathway in resource-scarce environments. Formal statistical tests were applied to compare regression coefficients across subgroups to confirm the significance of these disparities.

Fourth, we performed mediation analyses to decode the underlying pathways. To empirically weigh the “Primacy of We” against the “Primacy of I,” we estimated both parallel and serial mediation models. Given the potential non-normality of the data, we utilized bootstrap procedures with 5,000 resamples to generate bias-corrected 95% confidence intervals (CIs) for all indirect effects.

Finally, the sequential model specifically tested the path Physical Exercise → Confidence → Social Relationships → Happiness. This allowed us to clarify whether adult social benefits are a byproduct of improved self-efficacy or a direct result of communal participation.

## 4 Results

### 4.1 Descriptive statistics

The final analytical sample (*N* = 23,059) provides a robust foundation for examining the “Collective Resonance” model. As summarized in Table 1, the mean happiness score was 7.50 (SD = 2.06) on a 0–10 scale. High-frequency exercisers—those engaging in physical exercise (PE) at least once per week—accounted for 38.2% of respondents, while the majority (61.8%) were low-frequency or non-exercisers. Regarding our hypothesized mediators, the sample reported a mean confidence level of 4.10 (SD = 0.91) on a 1–5 scale, and social relationship quality averaged 7.04 (SD = 1.89). Demographic characteristics indicate a middle-aged population (Mean age = 42.50) with a significant representation of rural Hukou (73.4%) and employed individuals (80.3%).

**Table 1:** Variable definition and descriptive statistics (*n* = 23,059).

Category	Variable	Variable definition	Mean	Standard deviation
Dependent variable	Happiness	Self-rated level of happiness: 0 (‘very unhappy’) to 10 (‘very happy’)	7.50	2.06
Independent variable	Physical exercise	High-frequency exercisers (≥1 session per week) = 1 Low-frequency exercisers (<1 session per week or never) = 0	0.38	0.49
Mediating variables	Confidence	Self-rated confidence about future life: 1 (‘no confidence at all’) to 5 (‘very confident’)	4.10	0.91
	Social relationship	Self-rated level of social relationship with others: 0 (‘very poor’), 10(‘very good’)	7.04	1.89
Control variables	Age	Actual age of at survey	42.50	18.45
	Gender	Female = 0, Male = 1	0.51	0.50
	Health	1 = Very healthy to 5 = Unhealthy	2.77	1.19
	Marriage	Not married = 0, Married = 1	0.69	0.46
	Hukou (household registration)	Rural = 0, Urban = 1	0.27	0.44
	Employment	Not employed = 0, Employed = 1	0.80	0.40

Pearson correlation coefficients (see Table 2) provide preliminary evidence for the “upward spiral” of well-being. Physical exercise was significantly and positively associated with happiness (*r* = 0.098, *p* < 0.001), confidence (*r* = 0.019, *p* < 0.05), and social relationships (*r* = 0.061, *p* < 0.001). Notably, the moderate correlation between confidence and social relationships (*r* = 0.287, *p* < 0.001) suggests that internal psychological resources and external social resources may interact, though they represent distinct pathways to happiness. It is crucial to note that while these correlations reached statistical significance due to the exceptionally large sample size, the absolute magnitudes are small. In particular, the association between physical exercise and confidence (*r* = 0.019) is practically very close to zero, and should be explicitly characterized as a small but statistically significant association.

**Table 2:** Correlation matrix of key variables.

Variable	Happiness	Physical exercise	Confidence	Social relationship
Happiness	1.0000			
Physical exercise	0.0979*	1.0000		
Confidence	0.4197*	0.0187*	1.0000	
Social relationship	0.4735*	0.0606*	0.2867*	1.0000

*p < 0.05.

### 4.2 The direct returns: quantifying the association between exercise on happiness

To systematically test Hypothesis 1, hierarchical multiple linear regression analyses were conducted (see Table 3). To clarify the metric of our associations, the models report unstandardized regression coefficients alongside their standard errors. The hierarchical models were sequentially constructed to demonstrate the incremental explanatory power of each set of variables:

1. Model 1 (Control variables): Included only the demographic and socioeconomic control variables to establish a baseline. This model explained 6.0% of the variance in happiness (Adjusted *R*^2^ = 0.060).
2. Model 2 (Control variables + physical exercise): Introduced the core independent variable. This addition yielded a small but statistically significant increase in explanatory power (Adjusted *R*^2^ = 0.068; Δ*R*^2^ = 0.008, F-change *p* < 0.001). In this model, high-frequency physical exercise was positively associated with happiness (*B* = 0.371, *p* < 0.001), providing empirical support for H1.
3. Model 3 (Control variables + physical exercise + confidence): Added the first mediating variable, confidence. This step substantially increased the model’s explanatory power (Adjusted *R*^2^ = 0.208; Δ*R*^2^ = 0.140, F-change *p* < 0.001).
4. Model 4 (Control variables + physical exercise + confidence and social relationships): Incorporated both the internal (confidence) and external (social relationships) mediators into the full model. This final step further improved the model fit (Adjusted *R*^2^ = 0.337; Δ*R*^2^ = 0.129, F-change *p* < 0.001).

**Table 3:** Multiple linear regression analysis of factors influencing happiness.

Variables	Model 1	Model 2	Model 3	Model 4
Physical exercise	–	0.371*** (0.028)	0.313*** (0.026)	0.217*** (0.024)
Confidence	–	–	0.883*** (0.014)	0.653*** (0.013)
Social relationship	–	–	–	0.413*** (0.006)
Age	0.003** (0.001)	0.003** (0.001)	−0.001 (0.001)	−0.007*** (0.001)
Gender	−0.019 (0.027)	−0.036 (0.027)	−0.063* (0.025)	−0.038 (0.023)
Health	−0.452*** (0.012)	−0.443*** (0.012)	−0.281*** (0.011)	−0.222*** (0.010)
Marriage	0.216*** (0.034)	0.282*** (0.034)	0.072* (0.032)	0.142*** (0.029)
Hukou (household registration)	0.068* (0.030)	0.000 (0.031)	0.078** (0.028)	0.080** (0.026)
Employment	−0.148*** (0.037)	−0.120** (0.037)	−0.091** (0.034)	−0.166*** (0.031)
Constant	8.607*** (0.058)	8.368*** (0.061)	4.600*** (0.081)	2.758*** (0.079)
Adjusted *R*^2^	0.060	0.068	0.208	0.337
*N*	23,059	23,059	23,059	23,059

Unstandardized regression coefficients (B) are reported with standard errors in parentheses. ***p < 0.001, **p < 0.01, *p < 0.05.

Comparing the coefficients across the steps reveals the transition from direct to indirect associations. With the inclusion of both mediators in Model 4, the unstandardized coefficient for physical exercise decreased from 0.371 (in Model 2) to 0.217, but remained statistically significant (*p* < 0.001). This attenuation indicates an indirect association, providing initial evidence for the dual-path mechanism. Both mediators were strong positive predictors of happiness in the final model: confidence (*B* = 0.653, *p* < 0.001) and social relationships (*B* = 0.413, *p* < 0.001).

### 4.3 The geography of happiness: rural compensation and age-related resonance

To investigate the “Social Infrastructure” hypothesis, we stratified the sample to identify variations in the PE–happiness association (see Table 4).

**Table 4:** Results of heterogeneity tests.

Grouping variable	Subgroup	Coef.(B)	s.e.	*t*-value	*N*	*R* ^2^
Gender	Male	0.384***	0.039	9.73	11,676	0.080
	Female	0.348***	0.04	8.67	11,383	0.062
Age group	Youth (<45)	0.310***	0.036	8.68	12,339	0.102
	Middle-aged (45–59)	0.306***	0.061	4.99	6,075	0.090
	Elderly (≥60)	0.145*	0.067	2.18	4,645	0.065
Hukou	Urban	0.195*	0.046	4.21	6,143	0.076
	Rural	0.483*	0.034	14	16,916	0.085

***p < 0.001, **p < 0.01, *p < 0.05.

#### 4.3.1 Hukou status

A stark disparity emerged between urban and rural residents. PE had a markedly stronger association with happiness for rural residents (*β* = 0.483, *p* < 0.001) compared to urban dwellers (*β* = 0.195, *p* < 0.05). This supports the argument that in resource-scarce rural environments, collective exercise functions as a potentially important form of social infrastructure, providing a “social glue” that yields higher well-being returns.

#### 4.3.2 Age-related resonance

High-frequency PE significantly predicted happiness across all life stages: youth (*β* = 0.310), middle-aged (*β* = 0.306), and elderly (*β* = 0.145). The larger magnitude of association among younger and middle-aged adults suggests that the “building” of social and psychological resources via exercise is particularly potent during these high-stress life stages.

#### 4.3.3 Gender consistency

No statistically significant gender disparity was found (*p* = 0.890), indicating that the beneficial association between PE and happiness is a universal phenomenon in Chinese society, consistent across both males and females.

### 4.4 Decoding the pathways: the 2.2× multiplier of social relationships over confidence

It must be noted that because this study utilizes cross-sectional data, the following mediation results represent statistical mediations describing variance partitioned among variables, rather than temporal or causal psychological processes.

The hypothesized chain mediation model is a fully saturated model (df = 0) with only four observed variables (physical exercise, confidence, social relationships, happiness) and three freely estimated paths plus covariances. As is standard practice for just-identified/saturated mediation models estimated via bootstrap (Hayes and Rockwood, 2023; Preacher and Hayes, 2008; Barrett, 2007; Raykov et al., 2013), traditional global fit indices (e.g., CFI, RMSEA) are not meaningful and are not reported. Model fit is instead evaluated through the theoretical plausibility of path coefficients and the statistical significance and precision of bootstrapped indirect effects (5,000 resamples, bias-corrected CIs). All indirect effects reported below are based on this approach.

Before formally reporting the mediation results, it is worth noting that the bivariate correlations between physical exercise and confidence (*r* = 0.019, *p* < 0.05) and between physical exercise and social relationships (*r* = 0.061, *p* < 0.001) are relatively small. At first glance, this might appear inconsistent with the significant mediation effects observed in the subsequent regression and chain mediation analyses. This discrepancy is not uncommon in large-scale samples: regression-based estimates account for covariates and adjust for confounding factors, which can reveal relationships that are not apparent in simple correlations. Moreover, the large sample size (*N* = 23,059) provides sufficient statistical power to detect even small but meaningful effects. Therefore, although zero-order correlations are modest, the adjusted regression coefficients and bootstrap-based indirect effects reliably capture the psychological and social pathways through which physical exercise contributes to happiness.

To formally test Hypotheses 2–4, a sequential mediation model was estimated using bootstrap procedures with 5,000 resamples. Results, including bias-corrected confidence intervals, are presented in Tables 5, 6.

**Table 5:** Bootstrap analysis of mediating effect (parallel).

Effect type	Variable definition	Effect size	Boot SE	Bias-corrected 95% CI	Relative mediating effect (%)
Total effect	Physical exercise → Happiness	0.371	0.027	[0.318, 0.424]	
Direct effect	Physical exercise → Happiness	0.217	0.028	[0.177, 0.263]	58.49%
Total indirect effect		0.154	0.015	[0.126, 0.183]	41.51%
Indirect effect 1	Physical exercise → Confidence → Happiness	0.043	0.008	[0.028, 0.058]	11.59%
Indirect effect 2	Physical exercise → Social relationship → Happiness	0.111	0.011	[0.091, 0.133]	29.92%

**Table 6:** Bootstrap analysis of mediating effect (chain).

Effect type	Variable definition	Effect size	Boot SE	Bias-corrected 95% CI	Relative mediating effect (%)
Total effect	Physical exercise → Happiness	0.371	0.027	[0.318, 0.424]	
Direct effect	Physical exercise → Happiness	0.217	0.023	[0.172, 0.261]	58.49%
Total indirect effect		0.154	0.015	[0.126, 0.183]	41.51%
Indirect effect 1	Physical exercise → Confidence → Happiness	0.043	0.008	[0.028, 0.058]	11.59%
Indirect effect 2	Physical exercise → Social relationship → Happiness	0.096	0.010	[0.078, 0.118]	25.88%
Indirect effect 3	Physical exercise → Confidence → Social relationship → Happiness	0.015	0.003	[0.010, 0.020]	4.04%

#### 4.4.1 Parallel mediation: “we” vs. “I” (H2 and H3)

Consistent with our “Collective Resonance” model, social relationships emerged as the dominant mediator. The total effect of physical exercise on happiness was 0.371. The direct effect remained significant at 0.217, accounting for 58.49% of the total effect. The total indirect effect was 0.154, accounting for 41.51% of the total effect (see Table 5). A comparison of the two parallel paths reveals a significant hierarchy:

1. The Social Pathway: Indirect Effect via social relationships was 0.096, accounting for 25.88% of the total effect.
2. The Confidence Pathway: Indirect Effect via confidence was 0.043, accounting for 11.59% of the total effect.

This 2.2× multiplier of social relationships over individual confidence confirms H3, suggesting that the “social heart” of exercise is more critical for Chinese well-being than individual self-mastery.

#### 4.4.2 Serial mediation: the directness of adult integration (H4)

As presented in Table 6, the serial pathway (PE → Confidence → Social Relationships → Happiness) was statistically significant but substantively modest, accounting for only 4.04% of the total effect (Indirect Effect = 0.015, 95% CI [0.010, 0.020]). While H4 received empirical support, the small magnitude indicates that for Chinese adults, the social benefits of exercise are directly associated with communal participation rather than a secondary byproduct of improved self-image. Unlike adolescent-centric models, adult happiness is associated more with immediate social resonance rather than a developmental chain of psychological shifts.

#### 4.4.3 Summary of mediation findings

In summary, the results validate a hierarchical model of exercise-induced happiness in China. While exercise is linked to well-being through both internal and external resources, the “collective resonance” facilitated by social relationships exhibits a relative indirect effect more than twice as large as the individualistic path of confidence. Furthermore, the heightened association of exercise in rural areas reinforces the role of communal physical activity as a form of social infrastructure that mends the social fabric in modern China.

## 5 Discussion

The present study, utilizing a large nationally representative sample of Chinese adults (*N* = 23,059), provides substantial evidence that regular physical exercise—defined as at least once per week—is significantly associated with higher levels of happiness. This association is partially mediated by two distinct yet interrelated pathways: future-oriented confidence and the quality of social relationships. Crucially, our findings reveal a culturally nuanced pathway where the social pathway emerges as the dominant engine of well-being, offering a meaningful extension to existing psychological theories.

It is important to contextualize the magnitude of these findings. Given the exceptionally large sample size (*N* = 23,059), even very small effects can reach statistical significance. As indicated by the zero-order correlations (e.g., *r* = 0.098 between physical exercise and happiness) and the absolute effect sizes in the regression models, the substantive effects are relatively small. However, at a population level, even modest associations hold substantial practical relevance. The superiority of the social pathway over the individual psychological pathway suggests that public health interventions leveraging social group dynamics may yield broader aggregate benefits for societal well-being than interventions focused solely on individual mastery.

### 5.1 From “I” to “we”: the cultural architecture of collective happiness

The direct effect of regular physical exercise on happiness (*β* = 0.371) represents a moderate effect size according to Cohen’s (1988) guidelines. Consistently, the bivariate correlation (*ρ* = 0.098) indicates a detectable association that remains stable after adjusting for confounders in multivariate models. More importantly, mediation analyses reveal a pronounced “social multiplier” effect: the indirect pathway through social relationships accounts for 25.88% of the total effect, more than twice the contribution of individual confidence (11.59%). This finding highlights the central role of social relational resources in explaining exercise-related happiness.

This pattern diverges from many Western studies, in which internal mastery experiences and self-efficacy typically emerge as the primary mediating pathways linking exercise to well-being (e.g., White et al., 2018; McAuley et al., 2006). Because the present study did not include direct psychometric indicators of collectivism nor conduct a cross-cultural comparison, we propose as a plausible hypothesis that this divergence may reflect the collectivist cultural orientation of Chinese society (Hofstede et al., 2010; Oyserman et al., 2002). In such contexts, interpersonal harmony and network embeddedness are generally considered core components of the self-concept. Within such contexts, well-being is less tightly coupled with individual achievement and more strongly grounded in relational connectedness. However, this interpretation should be approached cautiously; Western and Chinese contexts are not strictly opposing categories, and definitive conclusions regarding these cultural distinctions would require direct cross-cultural comparative data.

We hypothesize that in China, physical exercise frequently involves communal settings. Although the dataset utilized in this study only measured exercise frequency and did not directly assess whether exercise was performed individually or in a group, the specific type of exercise, or the level of social interaction, broader socio-cultural observations suggest that practices such as community square dancing and morning tai chi are highly prevalent. These activities offer a plausible explanation for how physical activity might satisfy relatedness needs, which have been shown to be particularly salient within East Asian populations under Self-Determination Theory (Chen et al., 2015; Ryan and Deci, 2017). As a result, the social capital associated with exercise carries disproportionate weight in predicting happiness compared to more individualistic societies. This finding underscores the cultural contingency of the “resource-building” process proposed by Broaden-and-Build Theory and extends both this framework and Self-Determination Theory by highlighting the primacy of socially embedded pathways in collectivist settings.

### 5.2 The directness of maturity: why adults do not need confidence to connect

The serial pathway (exercise → confidence → social relationships → happiness) was statistically significant but substantively small, accounting for only 4.04% of the total effect. This contrast with adolescent samples, where chain-mediation models report stronger sequential effects (Yao et al., 2022; Guo et al., 2024), reflects a developmental shift in how social ties are maintained. While adolescent networks are highly sensitive to individual psychological resources like self-efficacy, adult social relationships are more strongly shaped by stable, role-based factors such as marriage and occupation. Furthermore, according to Socioemotional Selectivity Theory (Carstensen et al., 1999), as individuals age, their social motivation shifts from “knowledge acquisition” to “emotion regulation” and deepening existing ties. For adults, confidence is not a prerequisite for interacting with established family members or neighbors. Thus, once basic mastery is achieved through exercise, further increments in confidence yield diminishing returns for social engagement. This suggests that the motivational sequence of “competence → relatedness” proposed by SDT may weaken as social roles become institutionalized over the life course.

### 5.3 Breaking the atomization: exercise as social infrastructure in rural China

The association between exercise and happiness is markedly stronger among rural residents (beta = 0.483) compared to urban residents (beta = 0.195). In rural China, where formal organizational infrastructure may be diminished, group-based exercise functions as a rare and socially legitimate arena for maintaining interpersonal networks.

This disparity is also linked to the nature of physical activity. Unlike individualized urban fitness cultures, rural exercise is dominated by collective activities like ‘square dancing’ (Guangchangwu). These activities involve high levels of behavioral synchrony, which research indicates significantly enhances social bonding and a sense of ‘we-ness’ (Tarr et al., 2014). Therefore, rural exercise is linked to stronger social-bonding pathways, corresponding to a greater return on happiness. This supports the “substitutability” hypothesis in environmental psychology, where physical activity serves a compensatory role in socially impoverished settings (Stokols, 2018).

### 5.4 Theoretical integration and novel contributions

By simultaneously testing parallel and serial mediation, this study moves beyond additive approaches to illustrate that psychological and social resources are hierarchically organized in the Chinese adult population. Social relationships emerge as the primary channel, with confidence playing a smaller, largely direct role. These findings enrich the Broaden-and-Build framework by demonstrating that the upward spiral of positive emotions is more pronounced in collectivist contexts through the rapid accrual of social rather than individual resources.

### 5.5 Practical and policy implications

The dominance of the social pathway carries clear prescriptive implications. The dominance of the social pathway has clear implications for policy. Interventions aimed at enhancing happiness through physical activity should prioritize socially facilitated formats—such as community sports leagues and age-friendly public spaces—over individualistic approaches. In rural areas, local governments should integrate group exercise into social-governance structures. Given that only 38% of Chinese adults meet the once-weekly threshold, scalable, socially embedded programs represent a cost-effective public mental health strategy.

### 5.6 Limitations and future directions

Several important limitations must be acknowledged. First, the cross-sectional design precludes definitive causal inference or the establishment of temporal ordering; thus, the possibility of reverse causality remains (e.g., happier individuals may exercise more frequently). Second, physical exercise was measured using a single item based solely on frequency, resulting in a lack of data regarding exercise duration, intensity, or whether the context of the activity was individual or social. Third, dichotomizing the continuous exercise variable may have resulted in information loss. Fourth, key constructs including confidence, happiness, and social relationships were assessed using single-item measures. Fifth, “collective resonance” was not directly measured as a distinct psychometric variable, but rather proposed as a theoretical framework to interpret the findings. Sixth, the reliance on self-reported data introduces potential common method bias. Finally, despite adjusting for several covariates, residual confounding due to unmeasured variables cannot be ruled out, and CFPS complex survey design sampling weights were not fully incorporated into the present baseline structural equations. Future longitudinal research employing multi-item scales, objective accelerometry, and direct measures of social interaction during exercise is essential to validate these preliminary findings.

## 6 Conclusion: toward a model of collective resonance

In conclusion, this study provides compelling evidence that the path to happiness in China is paved with collective movement. Engaging in physical exercise at least once per week is consistently and positively associated with greater subjective well-being among Chinese adults. While exercise is positively linked to both internal psychological growth and external social integration, the pathways explaining this happiness are distinctly hierarchical.

Our analysis suggests a plausible hypothesis that the “Chinese happiness equation” is primarily supported by the social dimensions of exercise. The quality of social relationships functions as the dominant mediator, offering a happiness return more than twice as large as that of individual confidence. This “social multiplier” suggests that the rhythmic synchrony of group-based activities—from community square dancing to team sports—does more than just build muscle; it mends the social fabric and satisfies the deep-seated cultural need for relatedness.

Furthermore, this study highlights a critical developmental shift in how we connect. Unlike younger populations where confidence acts as a prerequisite for social engagement, Chinese adults experience a more direct form of social integration. The serial pathway from confidence to social ties is minimal in this national sample, indicating that the communal benefits of exercise are immediate and inclusive, rather than contingent upon personal self-assurance.

These findings call for a paradigm shift in public health strategy. To effectively enhance population-level happiness, interventions should move away from the “solo fitness” model and prioritize socially oriented exercise programs. By investing in “social infrastructure”—such as communal sports events and age-friendly public spaces—policymakers can leverage the power of collective resonance to combat atomization and build a more harmonious, resilient society. Ultimately, in the Chinese context, we do not just exercise to feel better about ourselves; we exercise to feel better together.

## Data Availability

Publicly available datasets were analyzed in this study. This data can be found at: https://cfpsdata.pku.edu.cn/#/home.

## References

[ref1] Bandura A. (1997). Self-efficacy: The exercise of control. New York, NY: Freeman..

[ref2] Barrett P. (2007). Structural equation modelling: adjudicating model fit. Personal. Individ. Differ. 42, 815–824. doi: 10.1016/j.paid.2006.09.018

[ref3] Bollen K. A. Stine R. A. (1992). Bootstrapping goodness-of-fit measures in structural equation models. Sociol. Methods Res. 21, 205–229. doi: 10.1177/0049124192021002003

[ref4] Buecker S. Simacek T. Ingwersen B. Terwiel S. Simonsmeier B. A. (2021). Physical activity and subjective well-being in healthy individuals: a meta-analytic review. Health Psychol. Rev. 15, 574–592. doi: 10.1080/17437199.2020.1760728, 32452716

[ref5] Bull F. C. Al-Ansari S. S. Biddle S. Borodulin K. Buman M. P. Cardon G. . (2020). World Health Organization 2020 guidelines on physical activity and sedentary behaviour. Br. J. Sports Med. 54, 1451–1462. doi: 10.1136/bjsports-2020-102955, 33239350 PMC7719906

[ref6] Carstensen L. L. Isaacowitz D. M. Charles S. T. (1999). Taking time seriously: a theory of socioemotional selectivity. Am. Psychol. 54, 165–181. doi: 10.1037/0003-066X.54.3.165, 10199217

[ref7] Chen G. Li Y. Xu J. Zhang X. (2024). Relationship between physical exercise and risk of depression: an assessment of intermediate variables through self-assessed health status and life satisfaction in the Chinese family panel study (CFPS) data. Acta Psychol. 246:104253. doi: 10.1016/j.actpsy.2024.104253 PMC11682662 39735591

[ref8] Chen B. Vansteenkiste M. Beyers W. Boone L. Deci E. L. Van der Kaap-Decder J. . (2015). Basic psychological need satisfaction, need frustration, and need strength across four cultures. Motiv. Emot. 39, 216–236. doi: 10.1007/s11031-014-9450-1

[ref9001] Cohen J. (1988). Statistical Power Analysis for the Behavioral Sciences (2nd ed.). New York, NY: Routledge., 11315248

[ref9] Demir Kaya M. Kaya F. (2026). What role do self-liking and self-competence play in the relationships between exercise identity and mental well-being in individuals who exercise regularly? BMC Psychol. 14:582. doi: 10.1186/s40359-026-04334-7, 41808170 PMC13097858

[ref10] Diener E. Inglehart R. Tay L. (2013). Theory and validity of life satisfaction scales. Soc. Indic. Res. 112, 497–527. doi: 10.1007/s11205-012-0076-y

[ref11] Diener E. Seligman M. E. P. (2002). Very happy people. Psychol. Sci. 13, 81–84. doi: 10.1111/1467-9280.00415 11894851

[ref12] Eime R. M. Young J. A. Harvey J. T. Charity M. J. Payne W. R. (2013). A systematic review of the psychological and social benefits of participation in sport for adults: informing development of a conceptual model of health through sport. Int. J. Behav. Nutr. Phys. Act. 10:135. doi: 10.1186/1479-5868-10-135, 24313992 PMC4028858

[ref13] Flora D. B. Curran P. J. (2004). An empirical evaluation of alternative methods of estimation for confirmatory factor analysis with ordinal data. Psychol. Methods 9, 466–491. doi: 10.1037/1082-989X.9.4.466, 15598100 PMC3153362

[ref14] Fredrickson B. L. (2001). The role of positive emotions in positive psychology: the broaden-and-build theory of positive emotions. Am. Psychol. 56, 218–226. doi: 10.1037/0003-066X.56.3.218, 11315248 PMC3122271

[ref15] Guo S. Fu H. Guo K. (2024). Effects of physical activity on subjective well-being: the mediating role of social support and self-efficacy. Front. Sports Act. Living 6:1362816. doi: 10.3389/fspor.2024.1362816, 38863568 PMC11165021

[ref16] Haslam C. Cruwys T. Haslam S. A. Jetten J. (2023). What explains the well-being benefits of physical activity? A mixed-methods analysis of the roles of participation frequency and social identification. Soc. Sci. Med. 340:116432. doi: 10.1016/j.socscimed.2023.116432 38052118

[ref17] Hayes A. F. (2022). Introduction to mediation, moderation, and conditional process analysis. 3rd Edn. New York, NY: Guilford Press.

[ref18] Hayes A. F. Rockwood N. J. (2023). Questions of value, questions of magnitude: an exploration and application of methods for comparing indirect effects in multiple mediator models. Behav. Res. Methods 55, 3772–3785. doi: 10.3758/s13428-023-02113-2 36253595

[ref19] Hofstede G. Hofstede G. J. Minkov M. (2010). Cultures and organizations: Software of the mind. 3rd Edn. New York, NY: McGraw-Hill.

[ref20] Iwon K. Skibińska J. Jasielska D. Kalwarczyk S. (2021). Elevating subjective well-being through physical exercises: an intervention study. Front. Psychol. 12:702678. doi: 10.3389/fpsyg.2021.702678, 34975608 PMC8719442

[ref9002] Liao T. Yin Y. Hu X. Tang S. Shim Y. (2023). The relationship between physical activity and subjective well-being in Chinese university students: the mediating roles of perceived health, social support and self-esteem. Front. Sports Act. Living 5:1280404. doi: 10.3389/fspor.2023.1280404, 37953863 PMC10634428

[ref21] Lubans D. R. Smith J. J. Morgan P. J. Beauchamp M. R. Miller A. Lonsdale C. (2018). Mediators of psychological well-being in adolescent boys. J. Adolesc. Health 62, 349–351. doi: 10.1016/j.jadohealth.2017.09.020, 26699231

[ref22] Mahindru A. Patil P. Agrawal V. (2023). Role of physical activity on mental health and well-being: a review. Cureus 15:e33475. doi: 10.7759/cureus.33475, 36756008 PMC9902068

[ref23] McAuley E. Konopack J. F. Motl R. W. Morris K. S. Doerksen S. E. Rosengren K. R. (2006). Physical activity and quality of life in older adults: influence of health status and self-efficacy. Ann. Behav. Med. 31, 99–103. doi: 10.1207/s15324796abm3101_14, 16472044

[ref24] Odacı H. Kınık Ö. Kaya F. (2026). The mediating role of self-esteem in the correlation between perceived parenting style and subjective well-being of young adults in university. Br. J. Guid. Couns. 54, 53–67. doi: 10.1080/03069885.2024.2429418

[ref25] Organization for Economic Co-operation and Development (2013). OECD guidelines on measuring subjective well-being. Paris: OECD Publishing. 24600748

[ref26] Oyserman D. Coon H. M. Kemmelmeier M. (2002). Rethinking individualism and collectivism: evaluation of theoretical assumptions and meta-analyses. Psychol. Bull. 128, 3–72. doi: 10.1037/0033-2909.128.1.3 11843547

[ref27] Peng W. Zeng H. Li J. (2023). The role of exercise identity and self-esteem in the relationship between physical activity and well-being: a longitudinal study of Chinese adolescents. J. Sports Sci. 41, 712–720. doi: 10.1080/02640414.2023.2223456

[ref28] Preacher K. J. Hayes A. F. (2008). Asymptotic and resampling strategies for assessing and comparing indirect effects in multiple mediator models. Behav. Res. Methods 40, 879–891. doi: 10.3758/BRM.40.3.879, 18697684

[ref30] Raykov T. Marcoulides G. A. Patelis T. (2013). Saturated versus just identified models. Educ. Psychol. Meas. 73, 1026–1033. doi: 10.1177/0013164412457367

[ref31] Resnick B. Inguito P. L. (2011). The resilience scale: psychometric properties and clinical applicability in older adults. Arch. Psychiatr. Nurs. 25, 11–20. doi: 10.1016/j.apnu.2010.05.001, 21251597

[ref32] Rhemtulla M. Brosseau-Liard P. É. Savalei V. (2012). When can categorical variables be treated as continuous? A comparison of robust continuous and categorical SEM estimation methods under suboptimal conditions. Psychol. Methods 17, 354–373. doi: 10.1037/a0029315, 22799625

[ref33] Ryan R. M. Deci E. L. (2017). Self-determination theory: Basic psychological needs in motivation, development, and wellness. New York, NY: Guilford Press.

[ref34] Stokols D. (2018). Social Ecology in the digital age: Solving complex Problems in a Globalized World. London, UK: Academic Press.

[ref35] Tarr B. Launay J. Dunbar R. I. M. (2014). Music and social bonding: “self-other” merging and neurohormonal mechanisms. Front. Psychol. 5:1096. doi: 10.3389/fpsyg.2014.01096 25324805 PMC4179700

[ref36] Teixeira P. J. Carraça E. V. Markland D. Silva M. N. Ryan R. M. (2012). Exercise, physical activity, and self-determination theory: a systematic review. Int. J. Behav. Nutr. Phys. Act. 9:78. doi: 10.1186/1479-5868-9-78, 22726453 PMC3441783

[ref37] Vella S. A. Aidman E. Teychenne M. Smith J. J. Swann C. Rosenbaum S. . (2023). The impact of sports participation on mental health and social outcomes in adults: a systematic review and meta-analysis. Syst. Rev. 12:113. doi: 10.1186/s13643-023-02288-937400880

[ref38] White R. L. Parker P. D. Lubans D. R. MacKinnon A. Astell-Burt T. Eime R. . (2018). Domain-specific physical activity and mental health: a meta-analysis. Am. J. Prev. Med. 55, 455–465. doi: 10.1016/j.amepre.2018.06.009 28153647

[ref39] Wiese C. W. Kuykendall L. Tay L. (2018). Get active? A meta-analysis of leisure-time physical activity and subjective well-being. J. Posit. Psychol. 13, 57–66. doi: 10.1080/17439760.2017.1374436, 37339054

[ref40] World Health Organization. (2020). WHO Guidelines on Physical Activity and Sedentary Behaviour. Geneva: World Health Organization. Available online at: https://www.who.int/publications/i/item/9789240015128

[ref41] Yao M. Ma Q. Liu X. Cao C. Lyu J. Guo W. (2022). The relationship between physical exercise and subjective well-being among Chinese junior high school students: a chain mediating model. Front. Psychol. 13:1053252. doi: 10.3389/fpsyg.2022.1053252, 36698574 PMC9868948

[ref43] Zhang K. Liu M. Yin Z. Kong N. Gong H. (2025). The effect of exercise motivation on adolescents’ subjective well-being: chain mediation of physical activity and social-emotional competency. BMC Public Health 25:1698. doi: 10.1186/s12889-025-22965-0, 40340715 PMC12060426

